# A quantitative evaluation of physical and digital approaches to centre of mass estimation

**DOI:** 10.1111/joa.12667

**Published:** 2017-08-15

**Authors:** Sophie Macaulay, John R. Hutchinson, Karl T. Bates

**Affiliations:** ^1^ Department of Musculoskeletal Biology Institute of Ageing and Chronic Disease University of Liverpool Liverpool Merseyside UK; ^2^ Structure & Motion Laboratory Department of Comparative Biomedical Sciences The Royal Veterinary College University of London Hatfield Hertfordshire UK

**Keywords:** biomechanics, centre of gravity, inertial properties, mass properties, validation, volumetric modelling

## Abstract

Centre of mass is a fundamental anatomical and biomechanical parameter. Knowledge of centre of mass is essential to inform studies investigating locomotion and other behaviours, through its implications for segment movements, and on whole body factors such as posture. Previous studies have estimated centre of mass position for a range of organisms, using various methodologies. However, few studies assess the accuracy of the methods that they employ, and often provide only brief details on their methodologies. As such, no rigorous, detailed comparisons of accuracy and repeatability within and between methods currently exist. This paper therefore seeks to apply three methods common in the literature (suspension, scales and digital modelling) to three ‘calibration objects’ in the form of bricks, as well as three birds to determine centre of mass position. Application to bricks enables conclusions to be drawn on the absolute accuracy of each method, in addition to comparing these results to assess the relative value of these methodologies. Application to birds provided insights into the logistical challenges of applying these methods to biological specimens. For bricks, we found that, provided appropriate repeats were conducted, the scales method yielded the most accurate predictions of centre of mass (within 1.49 mm), closely followed by digital modelling (within 2.39 mm), with results from suspension being the most distant (within 38.5 mm). Scales and digital methods both also displayed low variability between centre of mass estimates, suggesting they can accurately and consistently predict centre of mass position. Our suspension method resulted not only in high margins of error, but also substantial variability, highlighting problems with this method.

## Introduction

Centre of mass (CoM) is a fundamentally important anatomical and biomechanical parameter. At the level of the whole organism, it is a key determinant of stability at rest and in motion, and is therefore crucial in determining posture and limb kinematics (Gatesy & Biewener, 1991; Carrano & Biewener, 1999; Attwells et al. 2006; Young et al. 2007; Loverro et al. 2015). Knowledge of CoM and other mass properties (i.e. mass and moment of inertia) of individual body segments is also essential in determining how a whole organism can move. These mass properties are essential inputs in research seeking to quantitatively characterise the spatial translations and rotations of segments, the muscular forces required to achieve any given motion and the associated energetic costs (Kilbourne, 2013). As such, mass properties are primary input parameters in biomechanical approaches investigating locomotion using both inverse and forward dynamic assessments of movement. Through its consequences for segment movements and for the whole organism, CoM therefore has a highly significant impact on determining the locomotor capabilities of an organism, as well as its wider behaviours and ecological role. The impact of CoM on behaviours such as posture and locomotor capabilities has been assessed through various sensitivity analyses (Hutchinson, 2004b; Gatesy et al. 2009; Bates et al. 2010; Hobbs et al. 2014), which found CoM position to have a substantial impact on these traits, further highlighting the importance of accurate estimates of CoM. Given its fundamental importance, it is unsurprising that CoM position has been estimated in a variety of species, from primates and equids to dinosaurs (e.g. Sprigings & Leach, 1986; Crompton et al. 1996; Allen et al. 2009). Indeed, CoM is of particular interest in extinct taxa where it provides a valuable indirect route to information that cannot be directly observed. For example, on the locomotor habits of long extinct species, especially those possessing disparate body forms unlike those of living animals such as dinosaurs (e.g. Alexander, 1985; Henderson, 2004; Hutchinson et al. 2007; Sellers et al. 2013).

Historically, a range of physical methods have been employed to determine CoM position for whole organisms (e.g. Alexander, 1983, 1985; Henderson, 2003, 2006; Clemente, 2014), as well as organisms divided into their major component segments (e.g. Dempster, 1955; Sprigings & Leach, 1986; Crompton et al. 1996; Nyakatura et al. 2012; Andrada et al. 2013) (for overview, see Nigg & Herzog, 2007). Three primary physical methods are present in the literature, each having been applied to a range of species. Balancing approaches have been applied to whole organisms and to individual segments (e.g. Dempster, 1955; Dempster & Gaughran, 1967; Vilensky, 1979; Crompton et al. 1996; Myers & Steudel, 1997; Hutchinson, 2004a; Goetz et al. 2008). This has been done most frequently using forms of balance boards (Dempster, 1955; Vilensky, 1979), but also using knife edges (Goetz et al. 2008). Suspension techniques rely on the same physical principles, but involve the suspension of specimens (or body segments) from one point, where they are either allowed to hang naturally (Dempster, 1955; Dempster & Gaughran, 1967; Chandler et al. 1975; Fedak et al. 1982; Alexander, 1983, 1985; Rubenson & Marsh, 2009), or the position of the support is moved until they come to rest in alignment with a defined axis (Nauwelaerts et al. 2011). This process is repeated from multiple suspension points, from which results are overlaid (often with the help of photography, Fedak et al. 1982). The point of intersection of the lines of suspension then gives the CoM of the object under study. The third technique uses a scale, or scales, over which a specimen is supported to determine the moment arm of the specimen's weight that is acting on the scale at one end of the support system. Published variants of this approach include using scales at only one end of the system (Lephart, 1984; Sprigings & Leach, 1986; Walter & Carrier, 2002) or scales at both ends of the system with the organism lying on a support (Henderson, 2003; Kilbourne, 2013; Clemente, 2014) or resting directly on the scales (Henderson, 2006). It has been suggested that this technique is most effective when the CoM is only to be investigated along one axis at a time (Eshbach et al. 1990), though this is also the case for some variants of the suspension method. It should be noted that the balancing and suspension methods both work based on the same physical principles – that an object will only come to rest if it is supported through its CoM. In the case of suspension, an object left to hang freely will come to rest with its CoM in line with the string it is suspended from; i.e. the vector of its weight and the vector of tension in the string are parallel and collinear, passing through the CoM. In the case of balancing methods, a plate (and any object placed upon it) will only balance on a support if the combined CoM of the system lies directly above the support; i.e. the vector of combined weight, passing through the combined CoM of the system, passes through the support.

Very few studies investigating CoM position using physical methods such as those described above include any form of assessment of the accuracy of their methods. Although the physical principles behind each of the methods are sound, any physical experimentation method has the potential for error, at the very least human error, in the set‐up, capture and recording of data. Assessing the accuracy of the scale‐based technique, Lephart (1984) found mean absolute percentage errors of 0.03% in their estimations of CoM position (37 test objects ranging from 316 to 30 426 g, unknown geometries). The balance board technique employed by Sprigings & Leach (1986) resulted in a predicted CoM position within 2 mm of the geometric centre of their test object (an Olympic standard weightlifting disc: 20 kg, 450 mm diameter). Nauwelaerts et al. (2011) assessed the accuracy of their suspension method on test objects with simple geometries, finding that accuracy was dependent on the length and radius of their objects, and overall determined their method to be precise to within approximately 1 cm of the true CoM for a number of test objects of unknown geometries. Although some attempts have been made to compare results across studies (e.g. Nigg & Herzog, 2007), such comparisons are hindered by the often extremely limited descriptions of the methodologies used in many cases.

Recent advances in computing technology have seen digital modelling used more and more frequently as a method for calculating CoM position (e.g. Henderson, 1999; Hutchinson et al. 2007; Ren & Hutchinson, 2008; Amit et al. 2009; Bates et al. 2009a, 2016; Allen et al. 2013; Maidment et al. 2014; Park et al. 2014; Paxton et al. 2014; Nyakatura et al. 2015; Peyer et al. 2015). Digital models offer some advantages over physical methods including ease of data sharing and simple manipulation for sensitivity analyses and repeatability analyses, in addition to the advantages of scanning procedures such as computed tomography (CT). Models based on CT scans or similar data enable internal and external anatomy to be visualised and used as the basis for model generation, therefore incorporating a greater amount of the anatomical data available into models. It has been suggested that the detail of digital models is constrained more by researcher time than by technology limits (Allen et al. 2009), highlighting the extensive opportunities and challenges presented by this medium.

It is, however, frequently recognised that the validity of any methodology employing digital modelling techniques should be assessed before further application, and before any higher conclusions are drawn (Hutchinson, 2011). Comparisons are often made between physical measurements of body mass and values predicted from digital volumetric models (e.g. Henderson, 2006; Hutchinson et al. 2007; Allen et al. 2009; Bates et al. 2009a, 2015), where in some cases the discrepancies are appreciable (for example up to 16% in extant taxa, Allen et al. 2009). However, assessing the ability of a model to predict body mass accurately does not indicate how accurately the model is able to predict CoM. The CoM estimates produced by digital models are rarely checked (with some notable exceptions; e.g. Henderson, 2004, 2006; Hutchinson et al. 2007), in part due to the relative difficulty of physically measuring CoM in comparison with body mass. Considering the fundamental importance of CoM in biomechanical and functional analyses (e.g. for dinosaurs: Gatesy et al. 2009; Bates et al. 2010; Allen et al. 2013), and the ever‐increasing usage of digital models, the current lack of a comprehensive assessment of the accuracy of these digital modelling techniques in their ability to predict CoM is problematic.

This study aimed to assess the accuracy of three commonly used methods for estimating CoM by application to a set of objects with known geometries, as well as biological specimens. Two physical methodologies for CoM estimation and a digital volumetric approach were applied to each object. Due to the similarities between suspension and balancing methods, only one was investigated here. A version of the suspension method was selected over balancing for inclusion in this study because it did not require the fabrication of specialist equipment, and it has been more widely applied across disciplines and species, for example in studies of both extant (Dempster & Gaughran, 1967; Chandler et al. 1975; Fedak et al. 1982; Hutchinson et al. 2007; Nauwelaerts et al. 2011) and extinct (Alexander, 1985; Koehl et al. 2011) taxa. A variant of the scales method was also included. The comparison of results from these three methodologies to the geometric centres of the test objects enabled an assessment of absolute accuracy for each method. By comparison with each other, the relative accuracies of these commonly employed methods were then investigated. Application of each of these approaches to the same three biological specimens allowed absolute CoM predictions to be compared, in addition to enabling an examination of the differences in repeatability and logistical limitations between the methodologies.

## Methodology

### Specimens and background

Six specimens were studied here – three intact cadavers of birds (as specimens of biological interest) and three bricks. Bricks were selected due to their simple, known geometries, and therefore predictable CoM positons. The bricks acted as standards by which the absolute and relative accuracy of our methodologies could be assessed, and therefore aided our interpretation of the results obtained from biological specimens. The three birds studied here were a leghorn chicken (*Gallus gallus domesticus*, Leghorn), common buzzard (*Buteo buteo*) and mallard duck (*Anas platyrhynchos*), selected to represent a range of different avian body plans and locomotor types. The linear dimensions and masses of the six specimens studied here are presented in Table 1.

**Table 1:** Data on body mass and approximate dimensions for the six specimens studied here

Specimen	Mass (kg)	Dimensions (mm)^a^	Additional information
Brick1	3.13	216 × 99 × 67	
Brick2	2.37	214 × 102 × 65	
Brick3	4.26	203 × 210 × 133	
Chicken (*Gallus gallus domesticus*)	1.08	500 × 250 × 570	Leghorn chicken, male, 14 weeks
Buzzard (*Buteo buteo*)	0.69	475 × 230 × 980	Common buzzard, gender and age unknown
Duck (*Anas platyrhnchos*)	1.12	545 × 150 × 610	Mallard duck, female, age unknown

a imensions are listed along the axes EF × CD × AB (see Fig. 4 for more information on brick axes), and bird dimensions are listed along the axes cranio‐caudal × dorso‐ventral × left‐right.

As it was our aim to compare results from physical and digital methodologies, it was necessary to transfer the results of physical methods to digital space. This could be achieved in a variety of ways, such as a series of still photographs, or through the creation of photogrammetric models. Such methods have their merits (namely, that they are cheap and require no specialist equipment or knowledge to operate) and would be valid solutions to this problem. Here, however we opted to use an Oqus 7 Qualisys infrared motion capture system (www.qualisys.com), as the technology offered a quicker solution than photogrammetry, a more complete record of testing than still photographs and was not adversely affected by any movement of the specimens occurring during captures (e.g. during suspension testing). Calibration of the Qualisys system was performed before each data collection session to ensure that capture accuracy was suitably low, i.e. approximately 1 mm (mean error across cameras, across data collections: 1.32 mm). This represents an additional benefit over other potential methods, for which the error margins may be poorly investigated, and may vary considerably between trials. The Qualisys system used here consisted of 12 cameras, positioned around a large laboratory space usually used for capturing human gait trials. The Qualisys system enabled the 3D coordinates of a series of reflective spherical markers (12.7 mm diameter) to be captured and transferred to digital space. All raw data (including Qualisys and CT data captures), data at key stages of processing, and final data produced here are available online at http://datacat.liverpool.ac.uk/310.

**Figure 1:**
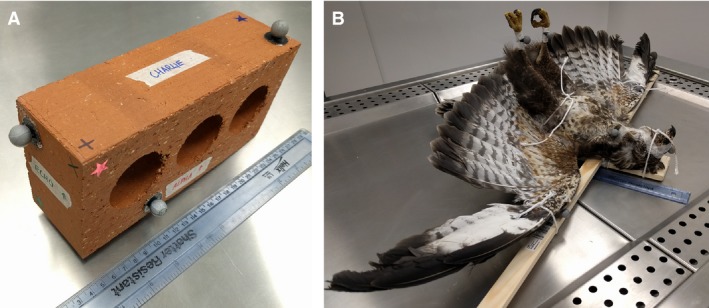
Pictures showing marker positions in bricks (A), and birds (B) as well as the standardised posture used for all bird specimens.

For the bricks, six markers were attached, one on each face (Fig. 1A). The faces were designated as A–F, as identification was essential for running later tests. Seven markers were attached to the birds in the following positions: cranial surface of the head, lateral surface of the torso at the junction with the neck, lateral surface of the torso at the junction with the tail, one on each wing tip (on the ventral and dorsal surfaces respectively; corresponding to the distal phalanges rather than the flight feathers) and two on the left and right distal tarsometatarsi (Fig. 1B). Markers were affixed to the skin (e.g. distal hindlimb) or to the outer surface of the birds’ feathers and secured with tape to minimise movement of markers between testing runs. In all cases, markers were placed to give maximal coverage of the whole object under study, including the geometric extremes.

Consistency of posture for the *ex vivo* bird specimens was crucial in preventing CoM shifts relating to postural changes, which could affect comparisons between methodologies (Allen et al. 2009). Before testing, all bird specimens were therefore thawed to enable them to be positioned in a standardised posture. The posture used here was selected to be comparable to those typically used in digital modelling studies (e.g. Allen et al. 2013), where the aim is to compare the morphology of often vastly different organisms, and is therefore unlikely to reflect a life‐like position used by any given specimen. Our standardised posture was as follows: head and neck fully straightened cranially, forelimbs straightened laterally as far as possible, hips extended as far as possible and the remaining hindlimb joints straightened as far as possible and allowed to freely hang ventrally (Fig. 1). To achieve this, specimens were tied to frames and then frozen at −20 °C. It was necessary to remove the frozen specimens from these supporting frames for the duration of each testing period. Some defrosting, and therefore postural changes, inevitably occurred during this time, with the extent dependent on the nature and duration of testing. Attempts were made to minimise any changes by packing specimens with ice for transport in the case of CT scanning, and replacing the specimens onto their frames after each testing phase. The magnitudes of these postural changes were quantified from data captured during testing. Posture change was measured by computing the 3D distance between each marker and each other marker for each testing condition. Inter‐marker distances were then compared across testing conditions; those with the largest summed discrepancy between tests were taken to represent the largest posture shift. Models from one of these testing conditions could then be manipulated to match the other extreme posture, thereby producing approximations of the CoM shift resulting from this change in posture. The resulting data informed the degree of caution necessary when drawing comparisons between testing methods for the bird data.

### Physical CoM – suspension methodology

**Figure 2:**
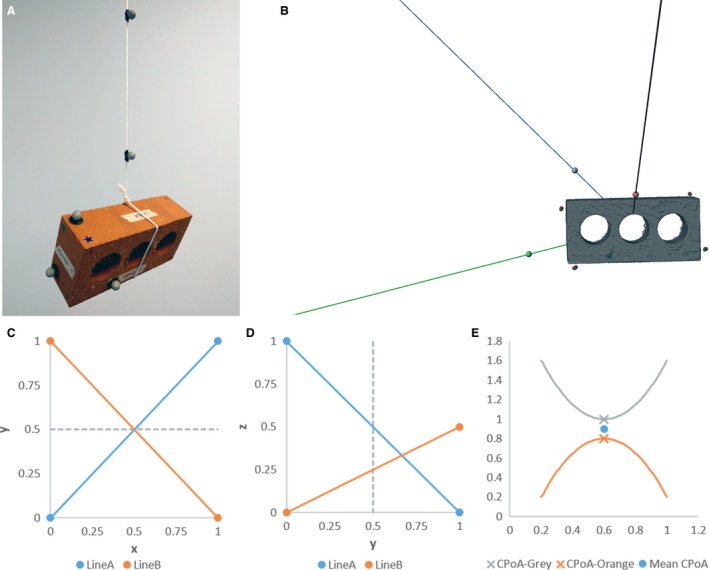
Stages of the suspension methodology performed in this study. (A) Suspension of object for Qualisys capture. At least three different suspension positions were captured for each object. (B) After the multiple Qualisys runs for the same specimen were matched, the specimen markers are aligned, and the various lines of suspension are now distributed around the specimen. (C,D) Two hypothetical 3D lines plotted in two, 2D graphs. Note that in (C), the lines have equal *x* values at *y* = 0.5, but at *y* = 0.5 in (D), they have different *z* values, and therefore do not intersect in 3D space. (E) Two hypothetical, non‐intersecting curves, highlighting the point of closest approach (CPoA) on each line, and the resulting mean CPoA.

Our suspension method is based on the approaches used by Alexander (1983) and Nauwelaerts et al. (2011). Specific details of our methodology follow, and a visual overview is presented in Fig. 2.

Specimens were suspended from a string via a loop that was tightened around each specimen. To estimate CoM with this method, it was also necessary to collect data on the position of the string in relation to the object under study. Two Qualisys markers were therefore placed on the string for each position, as far apart as possible (see Fig. 2A). The system was allowed to come to rest, after which a data capture was performed for at least 3 s (seconds) at 200 Hz. This procedure was repeated for multiple positions for each specimen. Between each position, specimens were removed from the string loop, repositioned and reaffixed to the string. Positions were selected attempting to provide coverage of the whole object, with at least one position taken in each plane. All specimens had data collected for at least three positions. To assess the impact of the selection of suspension location and other potential sources of human error on the CoM predicted by the method, data were collected for a total of 10 positions for one brick and two bird specimens.

The 3D marker coordinate data resulting from the Qualisys data captures for each position were exported, and marker coordinates from one timeframe extracted in matlab (www.mathworks.com). To determine CoM for each specimen, it was necessary to determine the point in space where the strings from each position intersected with one another in relation to the object. Coordinate sets for each object were therefore matched to one another, using the position of the markers directly on the specimens (i.e. those not on the string) as inputs. This was achieved using a global least square optimisation algorithm within the open source physics package gaitsym (www.animalsimulation.com). This algorithm matched the objects by a combination of translation and rotation in order to find the best global statistical fit (defined as the position with minimal error across all markers) between the two sets of markers. Once all the positions for a given object had been matched in digital space, the new coordinates for all markers were extracted in matlab.

The matched coordinates for the two string markers for each position were carried forward to estimate the overall object CoM. When considered in 2D (as in previous studies; e.g. Alexander, 1985), the point of intersection between two lines of suspension (here represented by the string) is the CoM estimated for that object. However, in 3D, two lines will very rarely intersect exactly with one another (see Fig. 2C,D for schematic representation of this). As an alternative to a strict intersect point, the point of closest approach was calculated for each pair of lines using custom matlab code, which is freely available online (http://datacat.liverpool.ac.uk/310). The mean of the two points of closest approach was taken to be the CoM predicted by those two lines (see Fig. 2E for simplified example). This approach was repeated for each pair of lines in turn, giving a total of three predicted CoM positions where three suspensions were carried out, and 45 predicted CoMs where 10 suspensions were carried out. The mean of all the predicted CoM positions for each object was then taken, giving the overall CoM predicted for that object by the suspension method (CoM_Su_).

### Physical CoM – scales methodology

**Figure 3:**
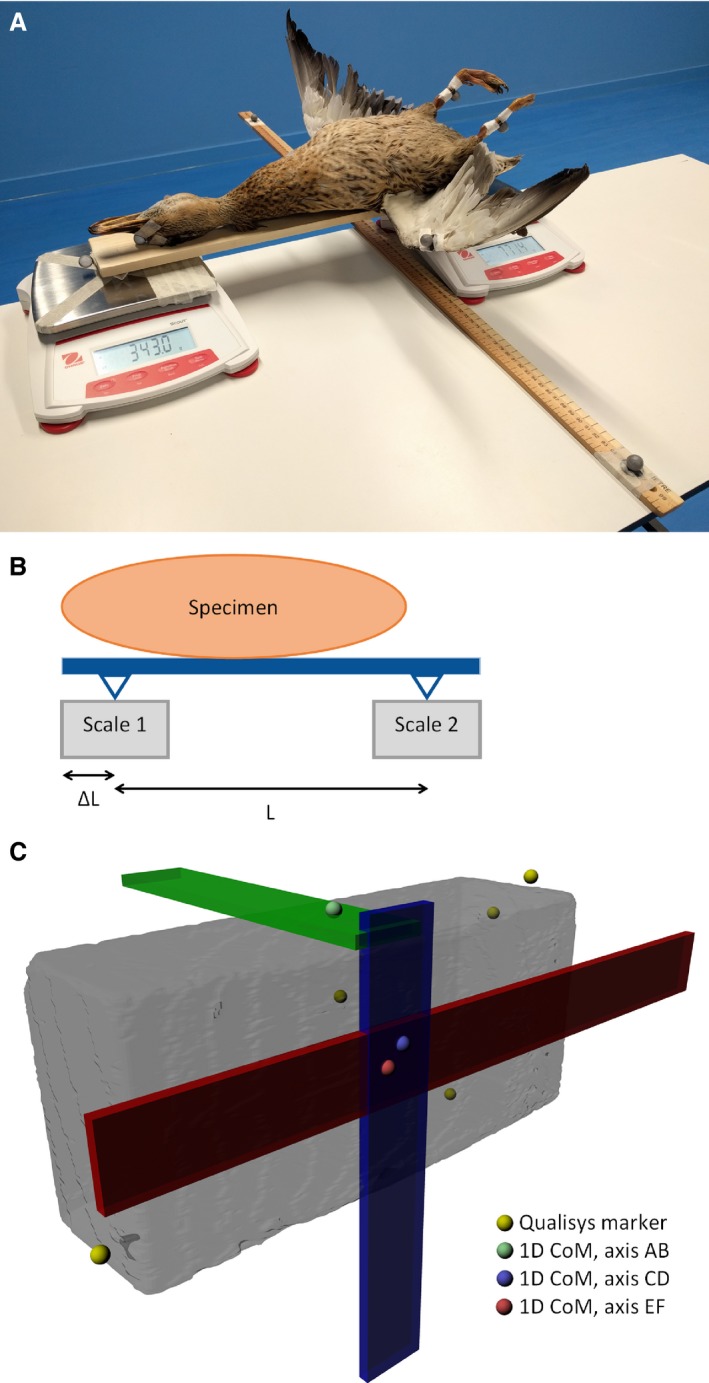
Stages of the scales methodology performed in this study. (A) Photograph of the experimental set‐up, with the duck specimen. (B) Schematic of experimental set‐up, showing specimen resting on plank, lying on the two scales. The distance between supports (*L*) and distance from proximal plank edge to proximal support (Δ*L*) are indicated. These data combined with mass readings from the two scales enable calculations of CoM position. (C) Rendering of Brick1, after marker data from three data captures was matched, showing the position of the three planks aligned with the three axes, and the three, 1D CoM positions plotted. These are then combined to give a 3D, final CoM prediction from the method.

Our scales method is based on the approach used by Clemente (2014). Specific details of our methodology follow, and a visual overview is presented in Fig. 3.

Two identical Ohaus Scout electronic balances (accurate to 0.1 g) were placed on a flat table. The scales were aligned with one another, and with the table they were resting on. A support was placed in the centre of each scale, perpendicular to the long axis of the table (Fig. 3A). Here, the support was an inverted triangular prism, length 60 mm. A wooden plank was then placed between the two supports so that it rested on them evenly, with care taken to align the plank with the long axis of the table (Fig. 3A). This plank, in addition to a metre ruler perpendicular to it, had one Qualisys marker placed at the centre of each end to enable the specimen‐scales system to be aligned to the digital world axes in the processing phase. The scales were positioned to give approximately 10–20 mm overhang between the plank ends and the centre of the supports (see Fig. 3A for experimental set‐up). Both scales were then tared.

The specimen under investigation was then placed onto the plank, with the proximal face of the brick, or the beak tip of the bird, in line with the proximal plank edge (see Fig. 3A for experimental set‐up). In the case of bricks, this was repeated for each of the three axes, with a different axis aligned with the plank in each run, in order to estimate CoM position in 3D. For bird specimens, CoM was measured only along the cranio‐caudal axis, resulting in 1D CoMs along that axis for all three birds. Although it is desirable to measure dorso‐ventral CoM position, to achieve this for the complex geometries of the biological specimens would have required specimen‐specific modifications to the experimental set‐up, which were deemed beyond the remit of this study. Although the cranio‐caudal axis is the primary axis of interest in many studies (e.g. Hutchinson, 2004a; Gatesy et al. 2009; Bates et al. 2010; Allen et al. 2013; Clemente, 2014), this represents a limitation of this methodology when applied to biological specimens where 3D CoM positions are essential to investigate problems in a complex 3D system such as an organism.

For each run, a Qualisys capture was performed (at least 3 s at 200 Hz), in addition to recording the values from the proximal and distal scales, the distance between the two supports, and the distance from the proximal plank edge to the centre of the proximal support. The distance of the CoM, along the axis that is in line with the plank, from the proximal plank edge could then be calculated as follows:

$${\text{CoM}}_{\mathrm{Sc}}=\left(W_{2}*\frac{L}{\left(W_{1}+W_{2}\right)}\right)+\Delta L,$$

where CoM_Sc_ is the distance of the CoM from the proximal plank edge along the axis of the plank, *W*_1_ and *W*_2_ are the masses on the proximal and distal scales respectively, *L* is the distance between supports and Δ*L* is the distance between proximal plank edge and the point where the proximal support contacts the proximal scale (see Fig. 3B for a schematic highlighting these values).

Runs for the same specimen were spatially aligned using gaitsym, as described in *Physical CoM – suspension methodology*. Bird and brick data were then plotted in maya (www.autodesk.com/maya). The specimen axes were aligned with the digital world axes using trigonometry based on the markers on the plank and ruler. Once aligned, calculated values for CoM_Sc_ could be plotted in digital space. In the case of bricks, it was necessary to plot three 1D CoMs, one for each axis investigated (see Fig. 3C). The combination of these 1D CoMs gave a 3D CoM for each brick, which along with the 1D CoMs for birds, formed the final coordinates for CoM_Sc_ for each specimen.

Using the original methodology described here, it was noted that the predicted CoM position was consistently skewed towards the proximal scale. To address this issue, ‘reversed repeats’ were conducted for two bricks. Here, a further three data collection runs were performed, so that each brick face was aligned with the proximal plank edge for one run, giving two runs per axis. Additionally, it was noted that the construction of this experimental set‐up and subsequent object placement had the potential to introduce human error into resulting predictions of CoM position. The associated error was therefore quantified using one brick, by conducting repeats where the experimental set‐up was de‐constructed and re‐constructed between each of five trials, with full data captures performed for each individual trial.

### Digital CoM – digital modelling

All specimens were scanned in a medical grade CT scanner at the University of Liverpool Small Animal Hospital, Leahurst (Toshiba Aquilion PRIME helical scanner, slice thickness: 1 mm, 120 kVp, 100 mA). All scan data are freely available online (http://datacat.liverpool.ac.uk/310). Scan data were segmented in avizo 7.1 (www.Avizo3D.com) using a combination of automated and manual thresholding as required to extract clean models. For bricks, the whole brick was extracted, along with the Qualisys markers. For birds, Qualisys markers, a solid skin outline, and all notable air cavities (defined as regions of zero density on CT scan) present in the torso, neck and head regions were extracted. The condition of the air cavities varied considerably between the bird specimens, due to differences in conditions and handling prior to freezing (Supporting Information Fig. S1). All air cavities were left as they were in the original frozen specimen, meaning the conditions captured in the CT scans were equivalent to those present during the experimental work, though they are unlikely to represent the resting condition for a living bird. Previous studies have shown that any subjectivity present in the segmentation process has minimal effect on the final mass properties estimated (Allen et al. 2009). To ensure our methodology followed this finding, segmentation of the original CT data for one brick was repeated, giving a total of three models.

Extracted surfaces were edited in geomagic studio 10 (www.geomagic.com) to remove any excess material captured by segmentation. Mass properties (volume and CoM) for the final surfaces were calculated in FormZ (www.formz.com). In the case of bricks, this CoM was the final digital CoM, but further steps were required for avian specimens due to the inclusion of multiple components (i.e. flesh and air cavities) in the models. For birds, the masses of each component were calculated from their respective volumes by the application of a density value of 1000 kg m^−3^, with air cavities subtracted where appropriate, as in numerous previous studies (e.g. Alexander, 1985; Henderson, 1999; Hutchinson et al. 2007; Allen et al. 2009; Bates et al. 2009a). This method is referred to as our ‘best guess’ digital CoM for birds, CoM_D1_. It should be noted that although this method is commonplace in recent literature, it represents a simplification of the anatomy, and one that has the potential to affect the CoM predicted by models. Though a thorough investigation of the consequences of these decisions on density modelling was beyond the scope of this study, a sensitivity analysis was conducted on this parameter to assess the impact on predicted CoM position. This was achieved by applying a range of published density data (Dempster & Gaughran, 1967; Tserveni & Yannakopoulos, 1988; Lovvorn & Jones, 1991; Buchner et al. 1997; Henderson, 2004, 2006), derived by a variety of methods in a range of taxa, to our models (see Table 2 for details). Once mass properties were calculated, centres of mass for all components were combined, to give an overall CoM for the specimen according to the following equation:

$${\text{CoM}}_{D}=\frac{\Sigma \left({\text{CoM}}_{f}*{\text{mass}}_{f}\right)-\Sigma \left({\text{CoM}}_{a}*{\text{mass}}_{a}\right)}{\Sigma {\text{mass}}_{f}-\Sigma {\text{mass}}_{a}},$$

where CoM_D_ is the digital CoM for the whole system, CoM_f_ and mass_f_ are the mass properties of flesh components, and CoM_a_ and mass_a_ are the air cavity mass properties.

**Table 2:** Details of the density data used in the sensitivity analysis

CoM abbreviation	Density data source	Taxonomic group	Density data applied (kg m^−3^)
CoM_D1_	Best guess (see e.g. Allen 2013)	Generic	Flesh: 1000, Air cavities: 0
CoM_D2_	Tserveni & Yannakopoulos (1988) – Homogeneous flesh (maximum density)	Bird	Flesh: 1069
CoM_D3_	Lovvorn & Jones (1991) – Homogeneous flesh (minimum density)	Bird	Flesh: 536.8
CoM_D4_	Henderson (2006)	Bird/archosaur	Head and Neck: 300, Trunk: 800, Limbs: 1000
CoM_D5_	Henderson (2003)	Bird/archosaur	Head: 1000, Neck: 600, Trunk: 850, Limbs: 1050
CoM_D6_	Dempster & Gaughran (1967)	Human	Head and Neck: 1170.8, Trunk: 1013.8, Forelimbs: 1080^a^, Hindlimbs: 1062^a^
CoM_D7_	Buchner et al.(1997)	Horse	Head: 1031, Neck: 1038, Trunk: 850, Forelimbs: 1155^a^, Hindlimbs: 1170^a^

a Values calculated as an average for all segments of that limb.

### Geometric centres

**Figure 4:**
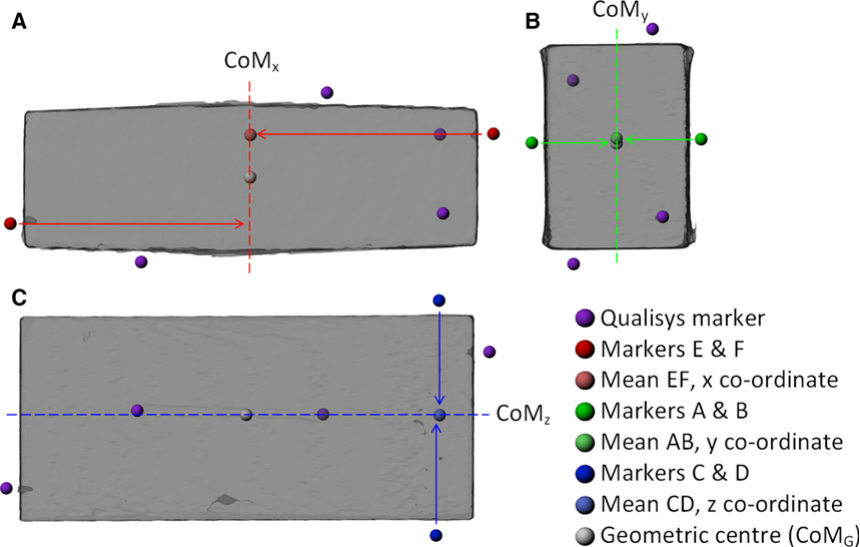
Render of Brick1 from the top (A), left side (B) and front (C) depicting the method for calculating the geometric centre (CoM_G_). This was calculated by taking the mean of three pairs of Qualisys markers, one pair per axis. CoM_x_, CoM_y_ and CoM_z_ then combine to give the final *xyz* co‐ordinates for a 3D CoM_G_.

For brick specimens, by virtue of their simple geometry, symmetry and uniform density, it was assumed that the geometric centre (CoM_G_) of each brick was also the true CoM position. The accuracy of each method could therefore be assessed by comparing the CoM predictions made with CoM_G_. CoM_G_ was calculated, after aligning bricks with the digital world axes as described in *Physical CoM – scales methodology*, by taking the mean coordinates of markers on opposite faces of the brick, using a different pair for each axis of interest. Combining these 1D coordinates gave the 3D CoM_G_ (see Fig. 4 for a visual overview).

To enable comparisons between methods, and with CoM_G_, the marker coordinates resulting from the two physical methods were matched to those extracted from the CT data of the corresponding specimen using gaitsym, as described in *Physical CoM – suspension methodology*. Once data from all methods were combined, models were translated so that, for the bricks, corner ADE (i.e. the corner shared by faces A, D and E) or the right hip (for birds) was at the origin of the world coordinate system in digital space (i.e. *x* = 0, *y* = 0, *z* = 0), for ease of interpretation of CoM values. All raw and processed data, as well as the code used to generate them, are freely available online (http://datacat.liverpool.ac.uk/310).

## Results

### Overview

**Figure 5:**
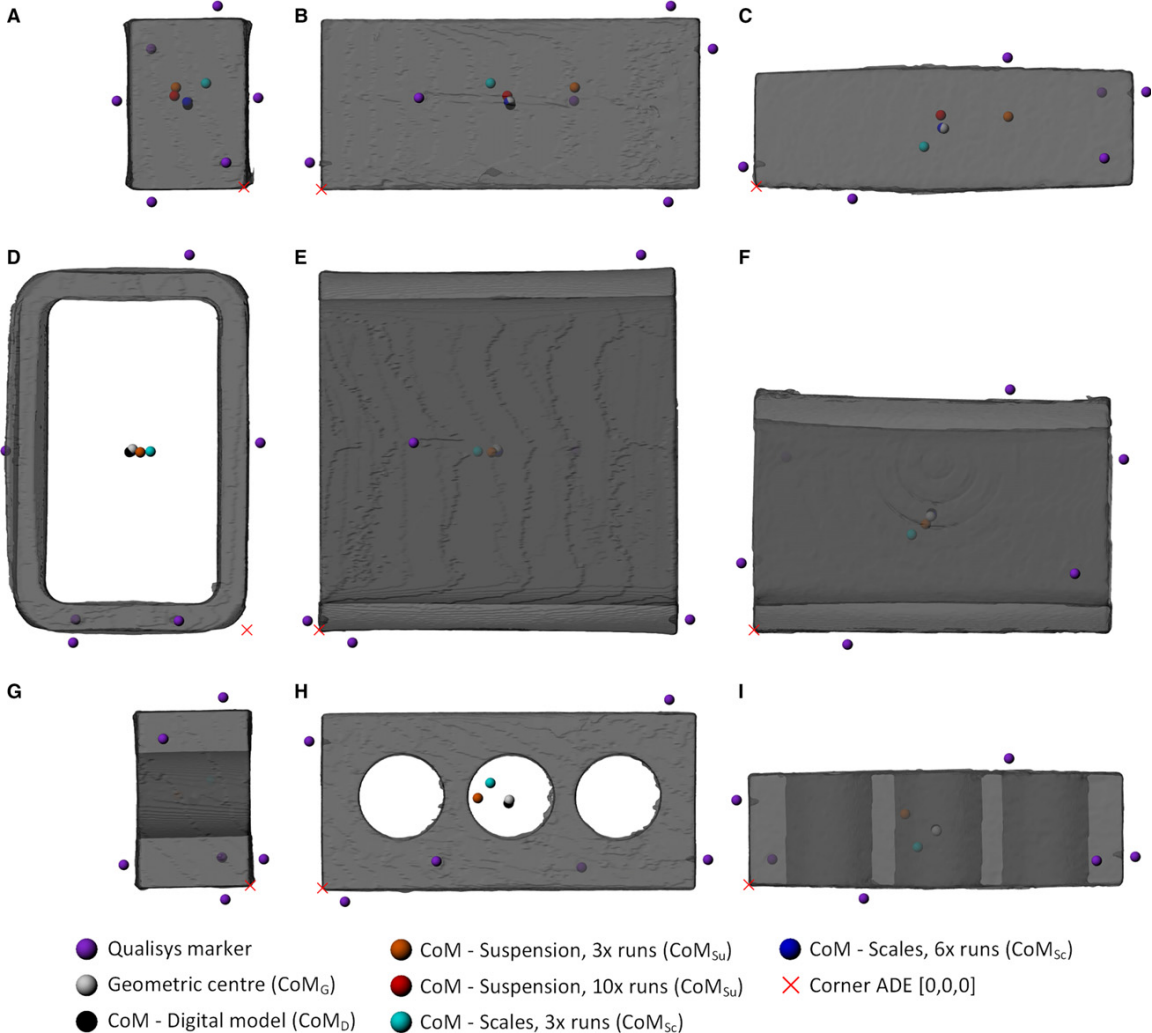
Predicted CoM positions displayed on renders of Brick1 (A–C), Brick2 (D–F) and Brick3 (G–I), shown from the left (A,D,G), front (B,E,H) and top (C,F,I). Predicted CoM positions are shown for each methodology, coloured according to the key. In cases where multiple CoM positions were available for the initial suspension and scales methods, only the CoM from the first runs are displayed here, for clarity.

**Figure 6:**
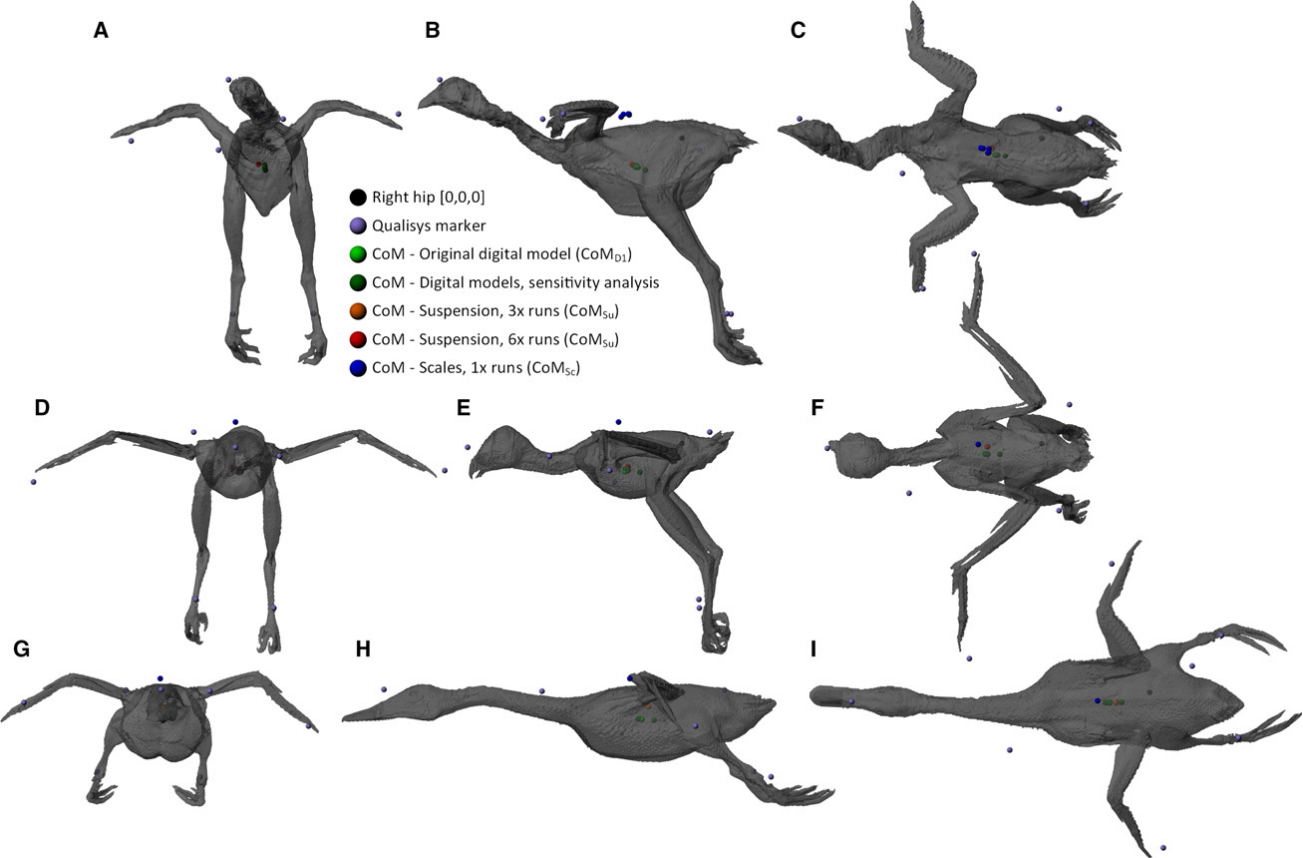
Predicted CoM positions displayed on renders of chicken (A–C), buzzard (D–F) and duck (G–I), shown in cranial view (A,D,G), left lateral view (B,E,H) and dorsal view (C,F,I). Predicted CoM positions are shown for each methodology, coloured according to the key. In the chicken and buzzard, multiple suspension CoMs are shown along with multiple scales CoMs in the chicken.

**Figure 7:**
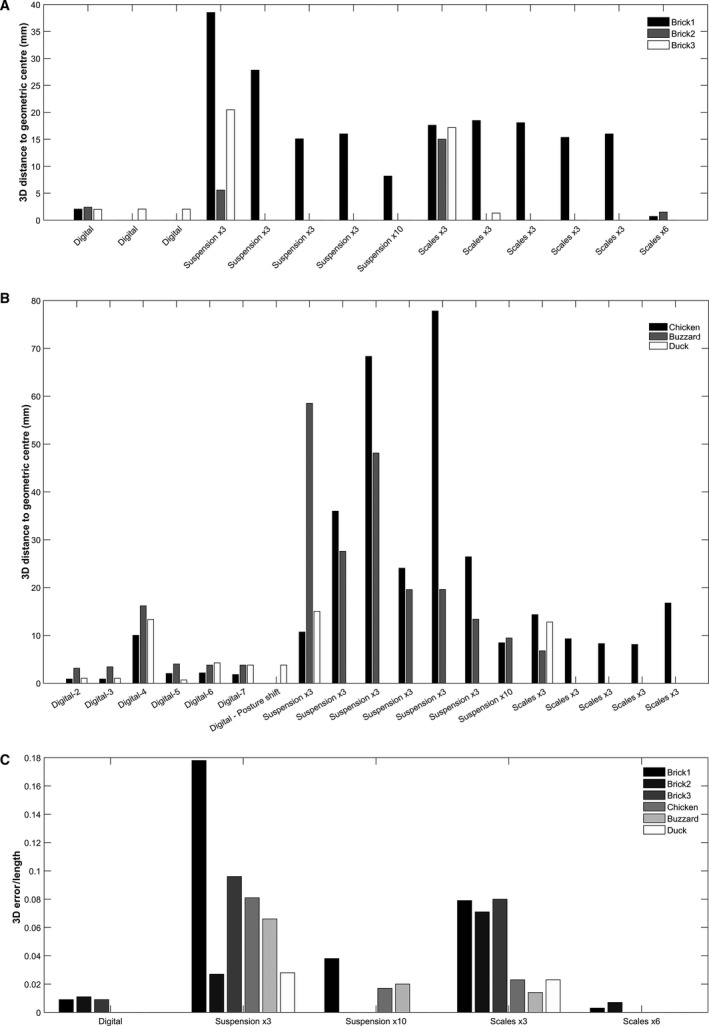
(A) Graph displaying 3D distances from brick geometric centre (CoM_G_) to the CoM positions predicted by the methodologies listed on the *x* axis. (B) Graph displaying 3D distances from our ‘best guess’ bird digital CoM (CoM_D__1_) to the CoM positions predicted by the methodologies listed on the *x* axis. (C) 3D differences between geometric centre (bricks)/best guess digital CoM (birds) and CoM predictions produced by the methods studied here, normalised by maximum side length (bricks)/cranio‐caudal body length (birds).

Data on the geometric centres and CoM positions predicted by each method conducted here are visualised in Figs 5 and 6, with differences presented in Fig. 7 and Tables 3 and 4. Further data on CoM positions are reported (Supporting Information Tables S1 and S2), along with data on 1D differences in CoM positions and normalised versions of CoM results (Supporting Information Figs S2 and S3). No statistics were performed on the data collected here; all results are therefore purely descriptive.

**Table 3:** 3D distances from brick geometric centre (CoM_G_) to the centres of mass predicted by the methodologies examined here for the three brick specimens

CoM description	3D distance to CoM_G_ (mm)		
	Brick1	Brick2	Brick3
Digital (CoM_D_)	2.1	2.4	2.0
Digital (CoM_D_)	–	–	2.1
Digital (CoM_D_)	–	–	2.0
Suspension (CoM_Su_) – 10 runs	8.2	–	–
Suspension (CoM_Su_) – 3 runs	38.5	5.6	20.5
Suspension (CoM_Su_) – 3 runs	27.8	–	–
Suspension (CoM_Su_) – 3 runs	15.1	–	–
Suspension (CoM_Su_) – 3 runs	16.0	–	–
Scales (CoM_Sc_) – 6 runs	0.7	1.5	–
Scales (CoM_Sc_) – 3 runs	17.6	15.0	17.2
Scales (CoM_Sc_) – 3 runs	18.5	–	–
Scales (CoM_Sc_) – 3 runs	18.1	–	–
Scales (CoM_Sc_) – 3 runs	15.4	–	–
Scales (CoM_Sc_) – 3 runs	16.0	–	–
Geometric (CoM_G_) – 3 runs	2.0	0.4	–
Geometric (CoM_G_) – 3 runs	0.4	–	–
Geometric (CoM_G_) – 3 runs	2.2	–	–
Geometric (CoM_G_) – 3 runs	0.9	–	–

**Table 4:** 3D distances from digital centre of mass predicted by our original model (CoM_D1_) to the centres of mass predicted by the methodologies examined here for the three bird specimens

CoM description	3D distance to CoM_D1_ (mm)		
	Chicken	Buzzard	Duck
Digital – CoM_D2_ – Tserveni (1988)	0.9	3.8	1.0
Digital – CoM_D3_ – Lovvorn (1991)	0.9	3.8	1.0
Digital – CoM_D4_ –Henderson (2006)	10.0	16.2	13.3
Digital – CoM_D4_ – Henderson (2003)	2.0	4.0	0.7
Digital – CoM_D6_ – Dempster (1967)	2.2	3.1	4.3
Digital – CoM_D7_ – Buchner (1997)	1.8	3.4	3.8
Digital – Extreme posture shift	–	–	3.8
Suspension (CoM_Su_) – 10 runs	8.5	9.5	–
Suspension (CoM_Su_) – 3 runs	10.7	58.5	15.0
Suspension (CoM_Su_) – 3 runs	36.0	27.6	–
Suspension (CoM_Su_) – 3 runs	68.3	48.1	–
Suspension (CoM_Su_) – 3 runs	24.1	19.6	–
Suspension (CoM_Su_) – 3 runs	77.8	19.6	–
Suspension (CoM_Su_) – 3 runs	26.4	13.4	–
Scales (CoM_Sc_) – 3 runs^a^	14.4	6.8	12.8
Scales (CoM_Sc_) – 3 runs^a^	9.3	–	–
Scales (CoM_Sc_) – 3 runs^a^	8.3	–	–
Scales (CoM_Sc_) – 3 runs^a^	8.1	–	–
Scales (CoM_Sc_) – 3 runs^a^	16.8	–	–

a Scales CoM positions in birds were only determined in one dimension, therefore the distances here are 1D only.

First, we report the results from bricks, stating the absolute and relative errors of the different methodologies in these reference objects in comparison with their known CoM positions. These results include those from the various repeatability tests. We then discuss results from bird specimens by methodology. It should be noted that, as true CoM is not known for the birds, the methodologies are instead compared back to our ‘best guess’ digital CoM predictions (CoM_D1_). The results reported for the avian specimens here are therefore strictly relative, suitable for comparison with one another, and do not provide a quantitative measure of the accuracy of any given method. Results from further repeatability tests are presented for these exemplar animals, which have more complex geometries and which therefore present more logistical challenges for testing than simple objects like bricks.

### Bricks

#### Geometric centres

The interpretations made here are reliant on the accuracy of estimates of the geometric centres (CoM_G_) of each brick. In an attempt to maximise the accuracy of CoM_G_, it was calculated using data from six runs wherever possible, or in the case of Brick3, from three runs. The variability present in CoM_G_ predictions from repeated runs was quantified in Brick1. When comparing CoM_G_ values for this brick, the difference between the alternative CoM_G_ predictions ranged from 0.374 to 2.18 mm, with a mean of 1.34 mm (Table 3).

#### Suspension method

Initial predictions of CoM by suspension (CoM_Su_) from three suspension positions were within 16, 5.6 and 20.5 mm of CoM_G_ for Bricks1–3, respectively (Table 3, Fig. 5). In Brick1, where data from four iterations of this basic suspension method were collected to assess the effect of human inputs, distance from CoM_Su_ to CoM_G_ ranged from 15.1 to 38.5 mm, a total range of 23.4 mm (Table 3, Fig. 5A–C). The error present in CoM_Su_ decreased when additional runs were performed on Brick1; for a total of 10 suspension positions, CoM_Su_ was then within 8.2 mm of CoM_G_ (Table 3, Fig. 5A–C). This represented a 66% improvement in the ability to predict CoM_G_ when 10, rather than three, positions were considered for Brick1 (Table 3, Fig. 5A–C). However, it should be noted that for Brick2, CoM_Su_ predicted from only three suspension positions was closer to CoM_G_ (5.6 mm; Table 3, Fig. 5D–F) than was CoM_Su_ for Brick1, predicted from 10 suspension positions (8.2 mm) (Table 3, Fig. 5A–C).

#### Scales method

Initial predictions of CoM by the scales method (CoM_Sc_) from three runs (one per axis), were within 17.6, 15 and 17.2 mm of CoM_G_, for Bricks1–3, respectively (Table 3, Fig. 5). The error present in CoM_Sc_ decreased substantially when additional ‘reversed repeats’ were performed (giving two runs per axis). This effect was assessed in Bricks1 and 2, where CoM_Sc_ was then within 0.691 and 1.499 mm of CoM_G_, respectively. Those ‘reversed repeats’ values respectively represented 90 and 96% improvements in the ability of the scales method to predict CoM_G_. Five CoM_Sc_ positions were predicted for Brick1 from repeats to assess the repeatability of this method, where the experimental set‐up had been completely deconstructed and reconstructed between runs. These predicted CoMs were between 15.4 and 18.5 mm from CoM_G_, a maximum variance of 3.13 mm (Table 3).

#### Digital modelling

Predictions of CoM position by the digital methodology (CoM_D_) were within 2.05, 2.39 and 2 mm of CoM_G_ for Bricks1–3, respectively (Table 3, Fig. 5). Two additional models were generated for Brick3 by repeating the segmentation of the raw CT data. For these additional repeats, CoM_D_ was within 2.05 and 2.03 mm of CoM_G_ (Table 3). The three CoM_D_ values estimated were highly consistent with one another, with a range of 0.058 mm.

#### Overview

Comparing the initial runs across the three bricks [i.e. three suspension positions, three scales captures (one per axis), and the initial CT segmentation], CoM_D_ was consistently closest to CoM_G_ (2.05, 2.39 and 2 mm; Fig. 7A). In Bricks1 and 3, CoM_Sc_ was the next closest (17.1 and 17.12 mm; Fig. 7A), followed by CoM_Su_ (24.4 and 20.5 mm; Fig. 7A). In contrast, in Brick2, CoM_Su_ was closer to CoM_G_ than was CoM_Sc_ (5.58 vs. 15 mm; Fig. 7A). The variation present in predicted values across bricks for these initial runs was lowest for CoM_D_ (0.391 mm), followed by CoM_Sc_ (3.47 mm) and CoM_Su_ (32.9 mm). Alternatively, considering only the best performing runs for each methodology [i.e. 10 suspension positions, six scales captures (two per axis), and the original CT segmentation] in Brick1, CoM_Sc_ was closest to the geometric centre (0.692 mm), followed by CoM_D_ (2.05 mm), with CoM_Su_ the most distant (8.18 mm).

### Birds

#### Suspension method

Initial predictions of CoM by suspension (CoM_Su_) in birds were within 10.7, 58.5 and 15 mm of CoM_D1_, for the chicken, buzzard and duck, respectively (Table 4, Fig. 6). As seen in the brick data, CoM_Su_ predictions were highly variable. In the chicken and buzzard, where six repeats of the basic suspension run were conducted, predicted values of CoM_Su_ varied by 67 and 45 mm, respectively (Table 4, Fig. 6A–F), a maximum distance of 77.8 mm from CoM_D1_. CoM_Su_ positions calculated from 10 runs were closer to CoM_D1_ than were those from three runs, for both the chicken (8.47  vs. 40.55 mm; Table 4, Fig. 6A–C) and the buzzard (9.46  vs. 31.1 mm; Table 4, Fig. 6D–F).

#### Scales method

Initial predictions of CoM position by the scales method (CoM_Sc_) were within 14.4, 6.8 and 12.8 mm of CoM_D1_ for the chicken, buzzard and duck, respectively (Table 4, Fig. 6). In the chicken, where the experimental set‐up was dismantled and reassembled between repeats, the variability between CoM positions was relatively low (8.66 mm; Table 4, Fig. 6), considerably lower than in suspension for avian specimens (i.e. up to 67 mm; Table 4, Fig. 6), but greater than that seen in equivalent repeats for bricks (3.13 mm; Table 4, Fig. 6).

#### Digital modelling

Digital CoM predictions in biological specimens require not only an accurate representation of object geometry (as for bricks), but also the assignment of density data. Results from the sensitivity analysis conducted on this variable show that the CoM predicted using density data from Henderson (2006) (CoM_D4_) was the most distant from the original CoM_D1_ in all three birds (10, 16 and 13 mm; Table 4, Fig. 6). The remainder of the CoM positions, predicted with applications of different density data (see Table 2 for details), were all close to one another, and to the original CoM_D1_ (maximum distance of 3.58 mm; Table 4, Fig. 6).

#### Quantifying posture change

The effect of posture change was quantified in the bird with the most extreme posture change between testing conditions (defined by the greatest total difference in distances between markers). Specifically, the greatest posture change occurred in the duck between the digital and suspension methodologies. The segments of the digital duck model were manipulated to match the Qualisys marker positions to their altered positions, as captured during the suspension runs. This rigid body transformation was achieved in maya by rotating segments around appropriate joint centres, indicated by the skeletal material. This resulted in a CoM shift of 3.81 mm from the original CoM_D1_ (Table 4). This can be considered an approximation of the maximum error present in CoM positions due to posture changes between the different methodologies. As the CoM positions predicted by the different methodologies in the biological specimens studied differed from one another by more than 4 mm, it can be concluded that the differences seen between methodologies are real and not the effect of postural changes between testing runs.

#### Overview

Taking the best runs from each methodology, CoM_Sc_ was marginally closer (8.11, 6.78 and 12.8 mm) to CoM_D1_ compared with CoM_Su_ (8.46, 9.46 and 15 mm), in these birds (Fig. 7B). It should be noted that the scales method used here did not include ‘reversed repeats’, which was shown to increase the accuracy of CoM predictions in the bricks (Table 3, Fig. 5). The variability within the methods showed similar trends to bricks: CoM_Su_ from three runs showed relatively high variability (up to 67.1 mm), CoM_Sc_ from three runs displayed relatively low variability (8.66 mm), and the variability seen in digital models with the sensitivity analysis on density parameters (if outlying data from Henderson, 2006 were excluded; see Discussion below) was lower again (1.28 mm).

## Discussion

### Overview

The CoM positions predicted by the three methodologies here varied considerably across each of the bricks (Table 3, Fig. 5). This variability is indicative of differences in their ability accurately to predict CoM_G_, which is taken to be a good measure of true CoM position (± 2 mm) in these test objects. For both bricks and birds, the variability present within methods was found to differ considerably between the three approaches (Tables 3 and 4, Figs 5 and 6). This is suggestive of differences in consistency and repeatability of the different methods. Briefly, we found that the scales methodology with reversed repeats was the most accurate, as well as being highly consistent (Tables 3 and 4, Figs 5 and 6). It was very closely followed by the digital method, which also appeared accurate, and with good consistency across the repeats performed on bricks here (Table 3, Fig. 5). The suspension method was identified as the least accurate, yielding predictions that were the most distant from CoM_G_, as well as displaying high variability between repeats (Tables 3 and 4, Figs 5 and 6).

We recognise that the small sample size studied here, and the associated lack of statistical testing has the potential to hinder conclusions being drawn about the differences between the methodologies investigated. However, we suggest that as the differences present between suspension and the other methods are so stark, and our experimental design identified and investigated multiple influencing factors, these descriptive results can be used confidently to identify real differences.

Though previous studies (Nauwelaerts et al. 2011) found correlations between features of object geometry and the error present in CoM predictions, we found no strong evidence of such an effect in any of the methods investigated here (Supporting Information Fig. S4). In the absence of a strong correlation, and given that all our objects are approximately equal in size, we suggest that the absolute errors reported here can be interpreted with confidence.

Normalising the data by length (longest side in bricks, cranio‐caudal body length in birds) did not change the overall trends seen in the data (Fig. 7C, Fig. S3). The digital and scales methods, including reversed repeats, still had very low errors (< 1.2% of body/brick length), further supporting our conclusion that these methods perform best for the specimens studied here. In the normalised data, errors relative to length were notably lower in birds than in bricks. This is a reflection of their greater lengths, and the fact that error is independent of size (Supporting Information Fig. S4).

Below, we discuss results from all methodologies in more detail, highlighting their benefits and limitations. We seek to identify issues with the methods, discuss the potential causes of these problems as well as possible solutions which could improve the future use of these methodologies.

### Suspension methodology

In the bricks, CoM_Su_ positions predicted from three runs were markedly different from CoM_G_ (3D distance: 15–38 mm), indicating this method performed relatively poorly at predicting CoM. This, along with the high variability (maximum range in bricks: 23 mm and birds: 67 mm) in the results not only indicates that this method is a relatively poor predictor of CoM_G_, but that there is also considerable variation in its ability to do so (Fig. 7). This is suggestive of complex human‐incurred error inherent to this methodology, with potential sources of error including the subjective selection of suspension position, and placement of string markers. A small amount of additional error was introduced here, as the string axis was defined using the raw marker centres, which were offset from the string itself (by 6.35 mm, the marker radius). However, this error was in one dimension only, and the effect was consistent across runs and between specimens. While this would affect the absolute accuracy of our suspension method, the error was small in comparison with the total error detected in this method (up to 38 mm), and did not affect our observation that results from the suspension methodology were highly variable. Further, the error present in this method was potentially influenced by the mass of the object under investigation. Error margins may be greater if an object of the same size as our bricks, but with a lower density, and therefore lower mass and inertia, is used as the test object. Such an effect may explain the different error margins seen in the brick and bird specimens, although we did not explicitly test this hypothesis.

**Figure 8:**
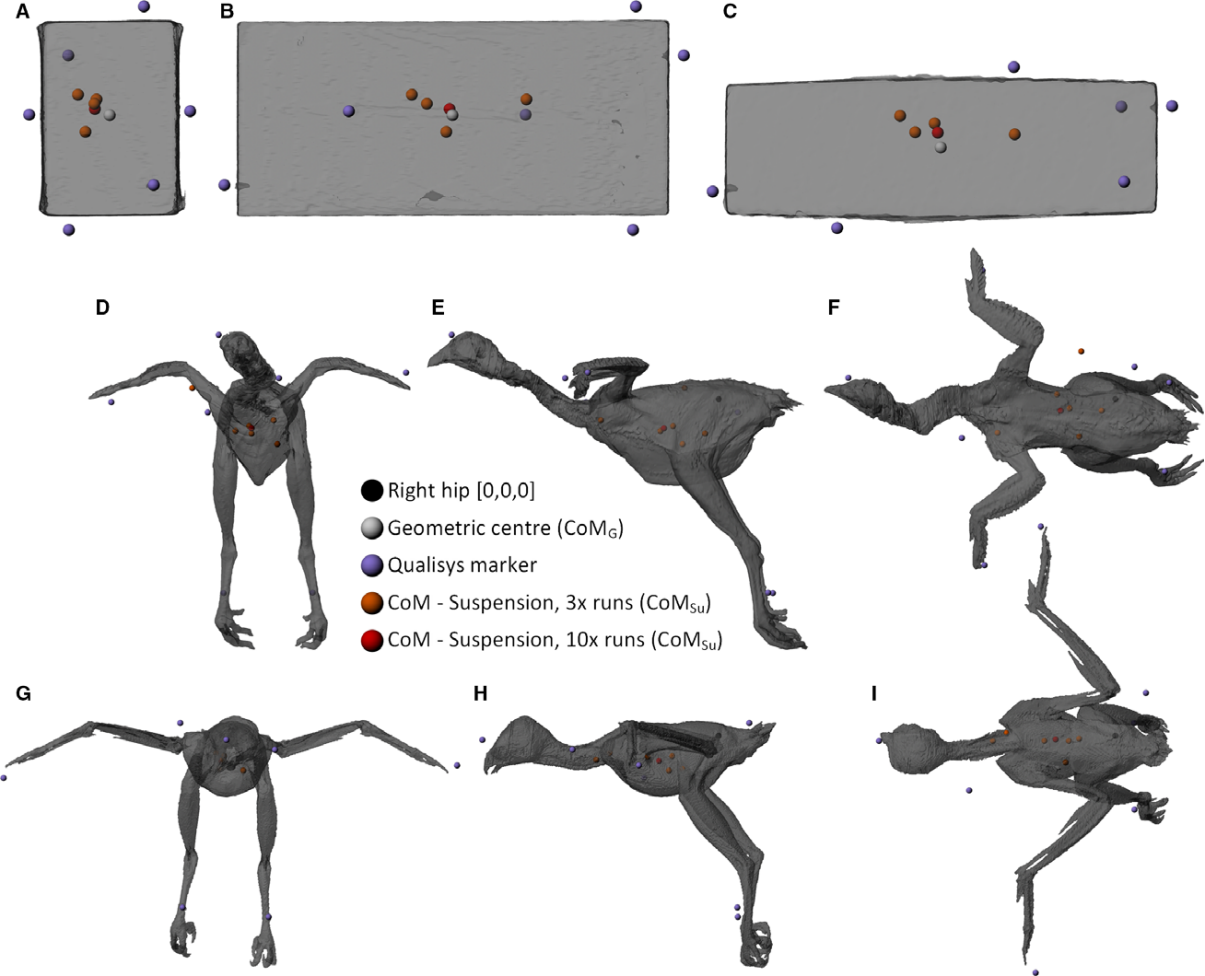
Renders of Brick1 (A–C), chicken (D–F) and buzzard (G–I) displaying the broad spread of centre of mass positions predicted by the suspension methodology with three repeats (orange) and 10 repeats (red).

For Brick1, the chicken and the buzzard, where additional suspension runs were conducted (taking the total to 10 runs, rather than three), the apparent accuracy of CoM_Su_ improved compared with the best results from three runs for those objects (Fig. 7). However, this improvement was only slight in the chicken (2.24 mm) and buzzard (3.93 mm). Additionally, the error in CoM_Su_ for Brick1 was actually higher than that obtained from only three runs on Brick2 (Fig. 7A). This further highlights problems with consistency in this methodology, regardless of the addition of further data runs. The addition of extra data captures here increases the number of unique lines of suspension (10 vs. 3) and therefore increases the number of string intersect points drastically (45 vs. 3). However, in the case of all the specimens studied here, these intersect points remained widely scattered (see Fig. 8). As the final predicted CoM_Su_ is calculated as the mean of all these points, the addition of more unique lines of suspension, and therefore intersect points, should act to increase the chances of a more central overall CoM being predicted, despite the fact that the accuracy of any given run does not improve. However, this is not a predictable effect and therefore results from three suspension runs may be more accurate than those from 10 runs. If enough unique suspension positions were tested, it might be possible consistently to derive highly accurate CoM positions from this methodology, but the cost in time and effort associated with performing the presumably large number of runs required might not always be desirable, particularly when other methods are available which address the issue in a more efficient, and more accurate manner.

### Scales methodology

CoM_Sc_ positions predicted from the original three runs for each brick were a notable distance from CoM_G_ (mean 3D distance of 17 mm, approximately equivalent to that in CoM_Su_). Despite this relatively low accuracy, the variation within these predictions was low (bricks: 3.13 mm, birds: 8.66 mm), indicating the relatively high repeatability of this method (Fig. 7). It was identified that there was a consistent shift of CoM towards the proximal scale; additional reversed repeats were conducted for two bricks in an attempt to counter this and to consequently improve the accuracy of this methodology. These repeats resulted in a drastic improvement in the ability of the scales method to predict CoM_G_ in bricks (within 0.69 and 1.49 mm in Bricks1 and 2, respectively; see Table 3, Fig. 7A). In both of those cases, the improved CoM_Sc_ was fractionally closer than CoM_D_ to the geometric centre of the brick (Table 3, Fig. 7A).

The short distance between CoM_G_ and CoM_Sc_ further highlights the absolute and relative accuracy of this methodology, provided that the appropriate repeats are conducted. It should be noted that in the birds studied here, only the initial runs (i.e. from the proximal end only, with no reversed repeats) were conducted. There is no reason to assume that the proximal skew observed in bricks would not also be seen in biological specimens. It is therefore safe to assume that the CoM_Sc_ positions predicted for birds are not accurate predictors of true CoM position, instead lying more cranially than the ‘true’ CoM position. The reversed repeats, which impart such a considerable improvement in CoM_Su_ prediction ability, are rarely conducted in the literature (with the notable exception of Henderson, 2003), with the vast majority of papers conducting only the initial runs performed here (e.g. Lephart, 1984; Kilbourne, 2013; Clemente, 2014). Our results suggest that reversed repeats are fundamentally important for this scale‐based methodology to predict accurately CoM position, and should therefore be employed wherever data on absolute CoM position are required from this method.

Investigation of the error associated with the subjective processes of de‐constructing and re‐constructing the scales experimental set‐up between data captures found relatively small errors (bricks: 3.13 mm, birds: 8.66 mm; Tables 3 and 4). However, it should be noted that this margin of error, along with that identified from the calculation of geometric centre in bricks (2.18 mm; Table 3), mean it is not possible confidently to distinguish between the accuracies of the scales and digital methods.

One key limitation of this scales methodology is the difficulty of deriving 3D CoM positions for biological specimens. Lying specimens along the plank was straightforward for the cranio‐caudal dimension here and could also be easily achieved for the medio‐lateral dimension (though the almost universal assumption of bilateral symmetry in analyses involving CoM limits the need to measure in this axis). However, determination of CoM position along the dorso‐ventral axis would require specimens to be positioned with that plane in line with the plank. While possible, developing a set‐up which would be capable of supporting a range of biological specimens in the precarious posture required, in a systematic and repeatable manner, was deemed to be beyond the scope of this study. Given that the scales method is accurate (to within 1.5 mm) along the cranio‐caudal axis, developing such a set‐up is an avenue that is potentially worth exploring. This is especially relevant for biological subjects, where the accuracy of digital CoMs are currently poorly constrained due to a scarcity of avian‐specific density data; a scale‐based method could therefore provide an avenue for validating digital CoM predictions in 1D or 2D. However, it should be noted that the error in the scales method (set‐up error, bricks: 3.13 mm, birds: 8.66 mm; error in CoM_G_: 2.18 mm; Tables 3 and 4) overlaps the error margin for digital estimates. This is the case for the cranio‐caudal axis, but the error present in an estimation of CoM along the dorso‐ventral axis is likely to be greater again due to the irregular shape of biological specimens. Hence overall error in CoM_Sc_ would be expected to exceed that present in a digital modelling approach when applied to biological specimens in more than one dimension. The relative merits and limitations of these techniques should be considered, along with specific aims of the study, when considering the best method to apply in future studies seeking to derive CoM estimates.

### Digital modelling

Predictions of CoM_D_ in bricks were close to CoM_G_ (3D distance: 1.99–2.39 mm; Table 3), indicating that the digital modelling method employed here resulted in accurate predictions of CoM position. Repeats of the segmentation protocol in bricks seeking to assess the variability introduced by that process found only minor differences (maximum difference between estimates: 0.39 mm; Table 3). Our findings therefore agree with those of Allen et al. (2009) obtained in biological specimens, that the process of digital segmentation from CT image data is highly repeatable, providing consistently accurate representations of object geometry which facilitate the accurate determination of CoM position.

In birds, however, an accurate representation of specimen geometry is only the first stage of digital model‐making. Biological specimens are heterogeneous, being composed of various tissue types with different densities, unlike bricks that can safely be assumed to be homogeneous. Previously, this heterogeneity has been recognised to a degree when constructing digital models, in order to provide a more realistic representation of not only volume distribution, but also of mass distribution. There is a history of including air cavities in digital models of birds and dinosaurs (e.g. Henderson, 1999; Hutchinson et al. 2007; Bates et al. 2009a). However, to our knowledge, the consequences of incorporating these structures have not been assessed to determine whether this brings predicted CoM closer to true CoM position. For other species (e.g. human, horse), more detailed mass properties are available on segment‐specific densities, which could be included in digital models. To our knowledge, there are no published data on segment‐specific densities for birds, and therefore the implications for CoM position of incorporating this additional heterogeneity are untested. We sought to explore the effects of this uncertainty with a sensitivity analysis here, applying our initial method, as well as five alternative sets of density data, to our bird models (Table 2).

Results from this sensitivity analysis show that five of the six density applications tested lie close to one another (within a maximum range of 3.58 mm across the three birds; Table 4, Fig. 7B). It is encouraging that the majority of data points cluster in this way, despite the use of a variety of density assignment methods, and the wide range of sources (including human and horse segment mass properties) for the density data applied. However, the CoM estimates generated using data from Henderson (2006) were markedly different from the others (10–15 mm from the main group, across the three birds; Table 4, Fig. 7B). The density values applied to the head and neck by Henderson (2006) seem unrealistically low (density: 300 kg m^−3^, cited as taken from Bramwell & Whitfield (1974); although it should be noted that we were unable to reconstruct this number from the original text, so this may be erroneous), and it is this low density which is the main contributor to the appreciably different CoM position predicted. The consistency of CoM predictions derived here using a range of density datasets highlights the relatively small effect of density variations on CoM position, provided broadly realistic data are used.

Application of different density datasets to different bird specimens resulted in different relative CoM shifts. The chicken and buzzard showed low variability, regardless of density data, with maximum CoM shifts of ~ 1 mm (Table 4, Fig. 6). The duck, however, displayed higher variability, with a maximum of 3.6 mm between CoM_D_ estimates (between CoM_D5_ in Henderson, 2003, and CoM_D6_ in Dempster & Gaughran, 1967) (Table 4, Fig. 6). This reflects a cranial shift in CoM_D_ when data from humans in Dempster & Gaughran (1967), and to a lesser extent horses in Buchner et al. (1997), are applied to the duck model. This difference is driven by differences in the neck and torso density values used in these studies. The fact that these differences appear in one bird and not the others, potentially reflects the different relative body proportions of these birds, which result in effects of different magnitudes by specific segments on the overall CoM. Alternatively, it may be indicative of different density datasets matching the true density data for some birds more closely than others. Unfortunately, no density data by segment are available for birds, nor is there a comprehensive quantitative examination of body proportions across Aves, so it is difficult to determine whether either or both of these, or indeed other factors, are influencing this trend.

## Conclusion

In conclusion, the scales (with reversed repeats) and digital modelling methods were found to be highly accurate predictors of true CoM position in the test objects examined here. The scales method was marginally more accurate (1.31 mm closer to CoM_G_; Table 3), though the error associated with calculating the geometric centres (up to 2.18 mm; Table 3) means the relative accuracies of these two methods cannot be confidently distinguished. Both scales and digital methods were identified as being highly consistent in their ability to predict CoM position, as well as demonstrating high levels of repeatability in experimental procedures. The suspension methodology was a generally poor predictor of CoM position, in addition to showing high variability and poor levels of repeatability (8.2–38.5 mm error; Table 3). These accuracies were assessed in test objects, with simple geometries and mass properties, and are arguably therefore a ‘best case’ representation of methodological accuracy. Biological specimens introduce additional complicating factors, varying by method. For the scales method, problems arise with the repeatability of capturing the required measurements; this is the case along the cranio‐caudal axis, but additional complications (and most likely greater error) would arise if data for additional axes were sought. Digital methods meanwhile face problems around the inclusion of heterogeneous densities. However, the sensitivity analysis conducted here, using a broad range of density datasets, found that variations in density data had a relatively low impact on CoM position. Provided bird segment densities do not differ substantially from the data used here, it is likely that uncertainty around density data will not introduce large inaccuracies in CoM position. However, we found that density has the potential to affect birds of different body plans differently, and there are currently no avian‐specific density data published to conclusively rule out density as an important influencing factor on CoM position. Future studies wishing to quantify CoM position in biological taxa should consider these factors in the light of their specific aims to determine the optimum method for CoM determination.

## Author contributions

Conceived project: S.M., K.T.B. Provided specimens: S.M., K.T.B. Collected data: S.M., K.T.B. Analysed and interpreted data: S.M. Drafted manuscript: S.M. Critically revised manuscript and approved article: S.M., J.R.H., K.T.B.

## Conflict of interest

The authors have no conflicts of interest to declare.

## Supporting information


**Fig. S1.** Renders of chicken (A,B), buzzard (C,D) and duck (E,F) showing the skin outlines (grey) and air cavities (blue) extracted from CT data and used in digital predictions of CoM position.
**Fig. S2.** Differences between geometric centre (bricks)/best guess digital CoM (birds) and CoM predictions produced by the methods studied here, presented as 1D differences for each axis.
**Fig. S3.** 1D differences between geometric centre (bricks)/best guess digital CoM (birds) and CoM predictions produced by the methods studied here, normalised by maximum side length (bricks)/cranio‐caudal body length (birds).
**Fig. S4.** Distance to geometric centre (i.e. error) plotted against side length, for three sides of three bricks of different dimensions.
**Table S1.** Data for centre of mass positions for three brick specimens, as predicted by the three different methodologies examined here.
**Table S2.** Data for centre of mass positions for three bird specimens, as predicted by the different methodologies examined here.
