# Supplementary material for: A quantitative evaluation of physical and digital approaches to centre of mass estimation
Source: J Anat. 2017 Aug 15;231(5):758–75. doi: 10.1111/joa.12667 (PMC5643916; doi:10.1111/joa.12667)
Supplement: Supplementary file 1 — Fig. S1. Renders of chicken (A,B), buzzard (C,D) and duck (E,F) showing the skin outlines (grey) and air cavities (blue) extracted from CT data and used in digital predictions of CoM position. Fig. S2. Differences between geometric centre (bricks)/best guess digital CoM (birds) and CoM predictions produced by the methods studied here, presented as 1D differences for each axis. Fig. S3. 1D differences between geometric centre (bricks)/best guess digital CoM (birds) and CoM predictions produced by the methods studied here, normalised by maximum side length (bricks)/cranio‐caudal body length (birds). Fig. S4. Distance to geometric centre (i.e. error) plotted against side length, for three sides of three bricks of different dimensions. Table S1. Data for centre of mass positions for three brick specimens, as predicted by the three different methodologies examined here. Table S2. Data for centre of mass positions for three bird specimens, as predicted by the different methodologies examined here. [file JOA-231-758-s001.pdf]

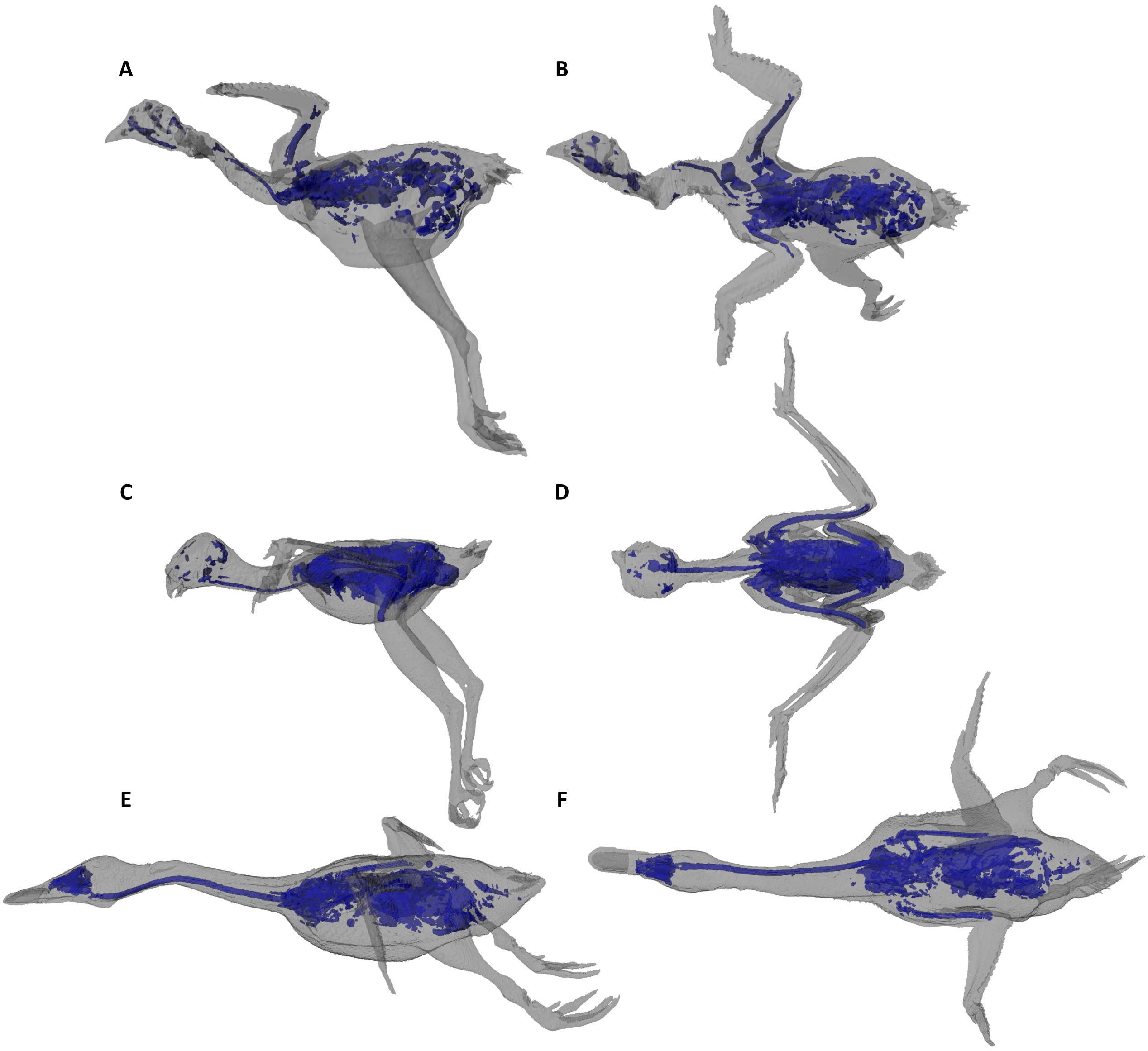

Fig. S1: Renders of chicken (A, B), buzzard (C, D) and duck (E,F) showing the skin outlines (grey) and air cavities (blue) extracted from CT data and used in digital predictions of CoM position.

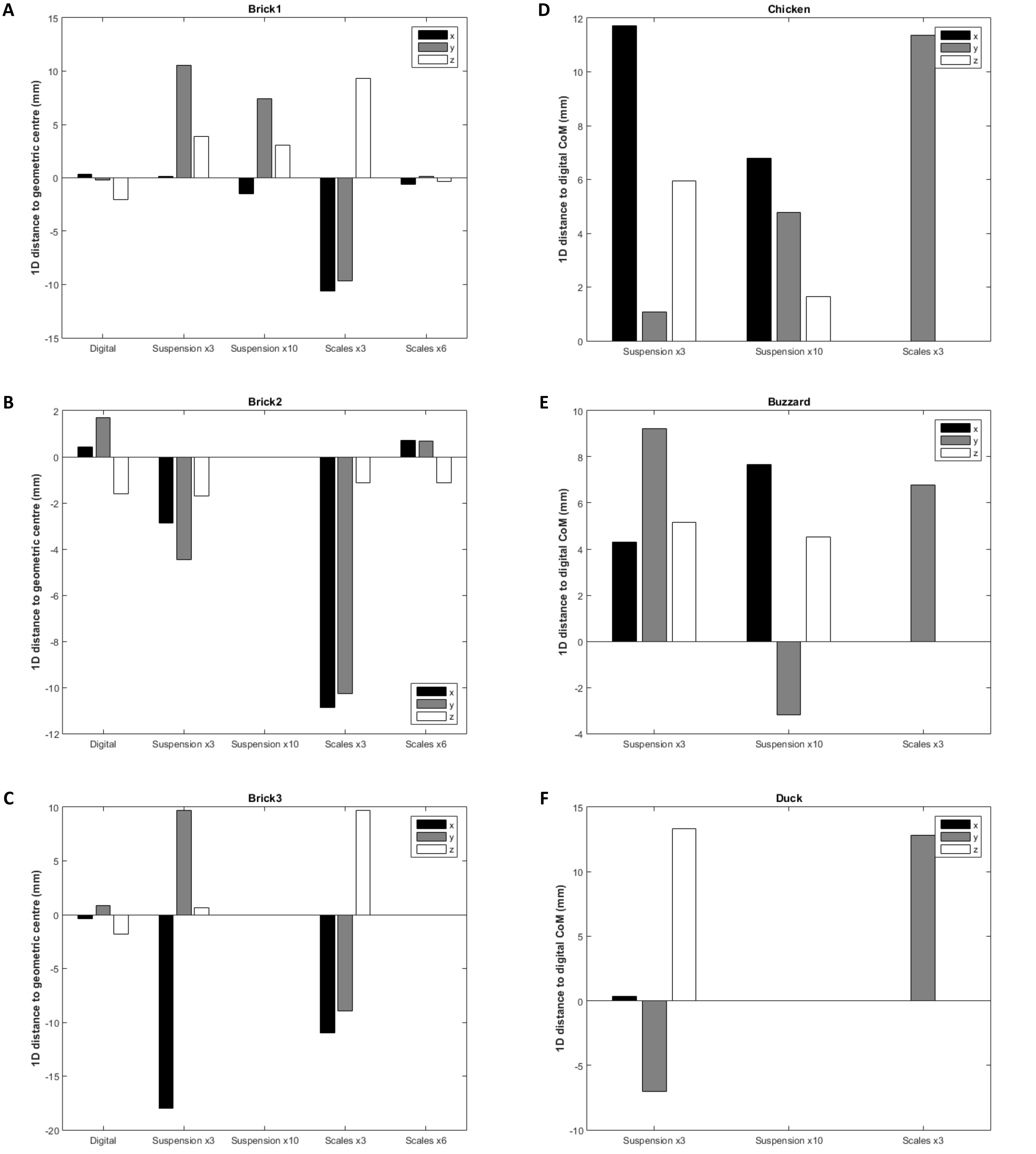

Fig. S2: Differences between geometric centre (bricks)/best guess digital CoM (birds) and CoM predictions produced by the methods studied here, presented as 1D differences for each axis. (A-C) Data for Bricks1-3, where positive values represent shifts towards sides F (x), B (y) and C (z) of the predicted CoM position relative to the brick geometric centre. (D-F) Data for chicken, buzzard and duck, where positive values represent right (x), cranial (y) and dorsal (z) shifts to predicted CoM position relative to the best guess digital CoM.

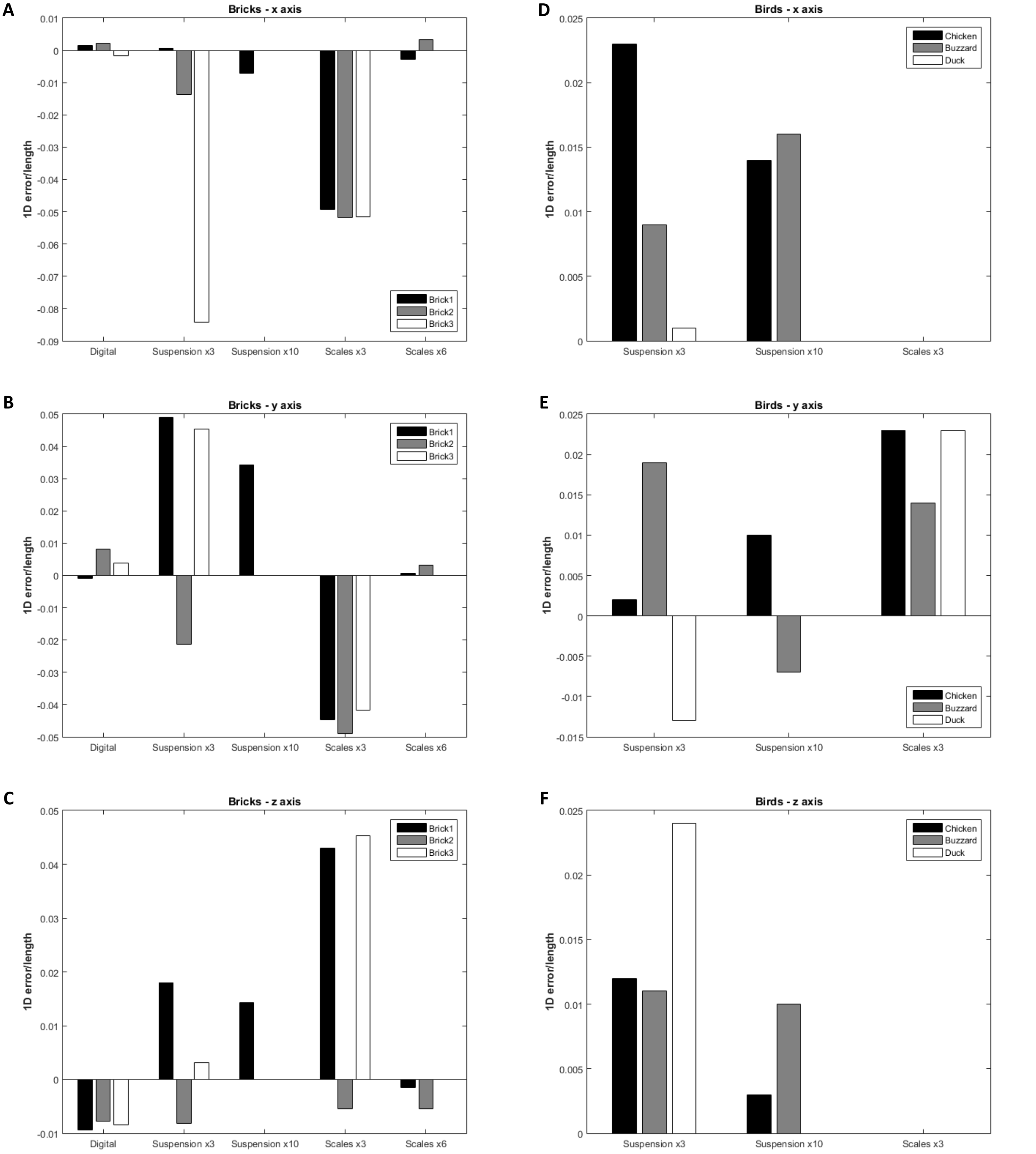

Fig. S3: 1D differences between geometric centre (bricks)/best guess digital CoM (birds) and CoM predictions produced by the methods studied here, normalised by maximum side length (bricks)/cranio-caudal body length (birds). (A-C) Data for Bricks1-3, presented by axis. (D-F) Data for chicken, buzzard and duck, presented by axis.

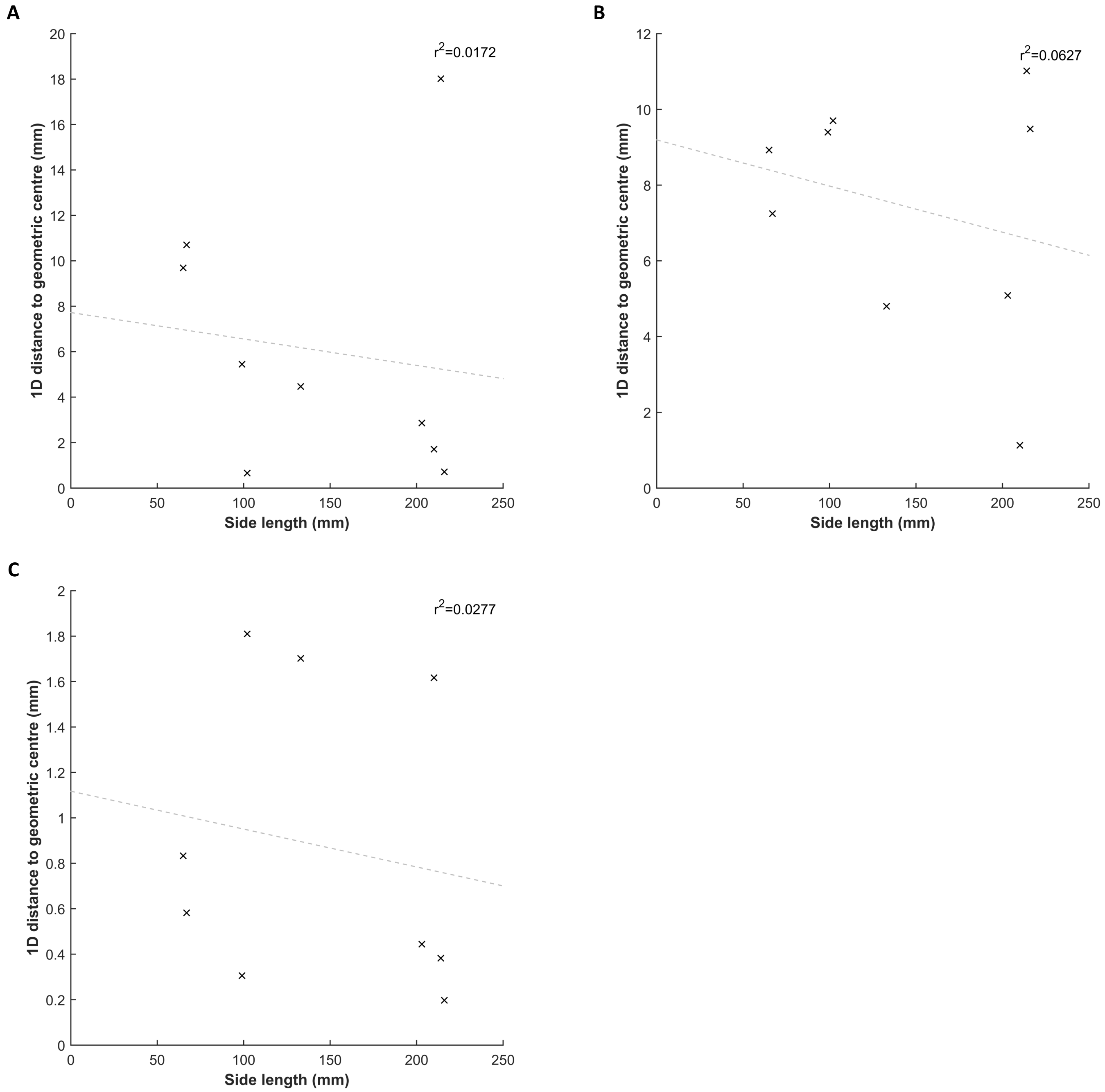

Fig. S4: Distance to geometric centre (i.e. error) plotted against side length, for three sides of three bricks of different dimensions. Least squares linear regression fitted to data, and r^2 value displayed at top right of each plot. For suspension (A), scales (B) and digital (C) methodologies.

Table S1: Data for centre of mass positions for three brick specimens, as predicted by the three different methodologies examined here. Where, x axis = E-F, y axis = A-B, z axis = C-D.

CoM Description	Brick1			Brick2			Brick3		
	x	y	z	x	y	z	x	y	z
Digital (CoM_D)	108.1	34.7	49.1	102.4	68.3	102.3	106.7	33.1	51.6
Digital (CoM_D)	-	-	-	-	-	-	106.8	33.1	51.5
Digital (CoM_D)	-	-	-	-	-	-	106.7	33.1	51.6
Suspension (CoM_{Su}) - 10 runs	106.3	42.3	54.2	-	-	-	-	-	-
Suspension (CoM_{Su}) - 3 runs	144.8	41.4	59.5	99.1	62.1	102.2	89.1	42.0	54.0
Suspension (CoM_{Su}) - 3 runs	87.3	50.7	61.2	-	-	-	-	-	-
Suspension (CoM_{Su}) - 3 runs	104.8	47.0	42.7	-	-	-	-	-	-
Suspension (CoM_{Su}) - 3 runs	94.9	42.5	56.7	-	-	-	-	-	-
Scales (CoM_{Sc}) - 6 runs	107.2	35.0	50.8	102.7	67.3	102.7	-	-	-
Scales (CoM_{Sc}) - 3 runs	96.4	25.5	60.8	91.1	56.3	102.7	96.1	23.3	63.1
Scales (CoM_{Sc}) - 3 runs	96.6	24.3	61.3	-	-	-	-	-	-
Scales (CoM_{Sc}) - 3 runs	96.2	25.0	60.8	-	-	-	-	-	-
Scales (CoM_{Sc}) - 3 runs	98.5	25.4	58.9	-	-	-	-	-	-
Scales (CoM_{Sc}) - 3 runs	98.2	25.8	60.2	-	-	-	-	-	-
Geometric (CoM_G) - 6 runs	107.8	34.8	51.1	102.0	66.6	103.9	-	-	-
Geometric (CoM_G) - 3 runs	108.3	34.1	49.4	102.1	67.0	103.6	107.1	32.3	53.4
Geometric (CoM_G) - 3 runs	108.1	34.9	51.0	-	-	-	-	-	-
Geometric (CoM_G) - 3 runs	108.1	34.8	49.0	-	-	-	-	-	-
Geometric (CoM_G) - 3 runs	108.2	35.1	50.4	-	-	-	-	-	-

Table S2: Data for centre of mass positions for three bird specimens, as predicted by the different methodologies examined here. Where, x axis = left-right, y axis = cranio-caudal, z axis = dorso-ventral.

CoM Description	Chicken			Buzzard			Duck		
	x	y	z	x	y	z	x	y	z
Digital - CoM_{D1} - Best guess	-18.1	48.4	-30.0	-11.2	61.2	-28.9	-11.1	43.1	-28.8
Digital - CoM_{D2} - Tserveni 1988	-18.9	39.4	-34.2	-11.4	45.0	-29.1	-10.5	29.8	-30.1
Digital - CoM_{D3} - Lovvorn 1991	-18.1	46.9	-31.3	-11.6	57.7	-27.0	-10.6	42.8	-29.1
Digital - CoM_{D4} - Henderson 2006	-18.0	48.8	-29.2	-11.2	58.5	-26.2	-11.0	44.1	-28.5
Digital - CoM_{D4} - Henderson 2004	-18.0	48.8	-29.2	-11.2	58.5	-26.2	-11.0	44.1	-28.5
Digital - CoM_{D6} - Dempster 1967	-17.8	50.4	-29.1	-11.5	60.6	-25.8	-10.8	47.3	-28.7
Digital - CoM_{D7} - Buchner 1997	-17.9	49.8	-31.2	-11.7	58.1	-27.6	-10.7	46.9	-28.7
Digital - Extreme posture shift	-	-	-	-	-	-	-14.7	44.2	-29.4
Suspension (CoM_{Su}) - 10 runs	-11.3	53.2	-28.4	-3.6	58.0	-24.3	-	-	-
Suspension (CoM_{Su}) - 3 runs	-9.0	44.4	-26.2	-5.0	119.1	-23.0	-10.8	36.1	-15.5
Suspension (CoM_{Su}) - 3 runs	-11.2	13.3	-33.5	-2.9	35.2	-32.5	-	-	-
Suspension (CoM_{Su}) - 3 runs	-32.8	114.5	-20.6	2.4	105.9	-17.4	-	-	-
Suspension (CoM_{Su}) - 3 runs	4.5	56.7	-31.0	-5.2	45.3	-19.3	-	-	-
Suspension (CoM_{Su}) - 3 runs	46.4	33.2	10.7	-26.0	48.4	-30.3	-	-	-
Suspension (CoM_{Su}) - 3 runs	-36.3	35.1	-43.8	-5.1	69.0	-19.8	-	-	-
Scales (CoM_{Sc}) - 3 runs *	-11.1	62.8	25.5	-0.9	68.0	23.5	-9.0	55.9	14.6
Scales (CoM_{Sc}) - 3 runs *	-16.0	57.7	25.9	-	-	-	-	-	-
Scales (CoM_{Sc}) - 3 runs *	-15.7	56.7	25.7	-	-	-	-	-	-
Scales (CoM_{Sc}) - 3 runs *	-11.6	56.6	24.9	-	-	-	-	-	-
Scales (CoM_{Sc}) - 3 runs *	-10.7	65.2	22.6	-	-	-	-	-	-

*Scales CoM positions were only determined in one dimension, along the cranio-caudal (y) axis.
